# 

**Published:** 1987-11

**Authors:** 


RISTOL MEDICO-CHIRURGICAL

OURNAL

### BRISTOL MEDICO-CHIRURGICAL JOURNAL

VOLUME 102(iv) NOVEMBER 1987

A National doctor morbidity Survey
General practice and Hospital Medicine—a new relationship
Predictive index for D.V.T.
From our correspondents
Dysphagia
Humour
Reports of Societies—
Gastro–enterology,
Clinical Pathology,
Orthopaedics

NATIONAL LIBRARY
APR 06 1988
OF MEDICINE

IN ASSOCIATION WITH

BRISTOL MEDICINE

THE MEDICAL JOURNAL OF THE SOUTH WEST

